# *Pmr-1* gene affects susceptibility of *Caenorhabditis elegans* to *Staphylococcus aureus* infection through glycosylation and stress response pathways' alterations

**DOI:** 10.1080/21505594.2019.1697118

**Published:** 2019-11-27

**Authors:** Emily Schifano, Graziella Ficociello, Simone Vespa, Salil Ghosh, John F Cipollo, Claudio Talora, Lavinia Vittoria Lotti, Patrizia Mancini, Daniela Uccelletti

**Affiliations:** a Department of Biology and Biotechnology “Charles Darwin”, University of Rome, Rome, Italy; b Department of Experimental Medicine, University of Rome, Rome, Italy; c Center for Biologics Evaluation and Research, Food and Drug Administration, Silver Spring, Maryland, USA; d Department of Molecular Medicine, Sapienza University of Rome, Rome, Italy

**Keywords:** Ca^2+^ATPase, *Caenorhabditis elegans*, glycosylation, infection, pathogens, *Staphylococcus aureus*

## Abstract

Calcium signaling can elicit different pathways involved in an extreme variety of biological processes. Calcium levels must be tightly regulated in a spatial and temporal manner in order to be efficiently and properly utilized in the host physiology. The Ca^2+^-ATPase, encoded by *pmr-1* gene, was first identified in yeast and localized to the Golgi and it appears to be involved in calcium homeostasis. PMR-1 function is evolutionary conserved from yeast to human, where mutations in the orthologous gene ATP2C1 cause Hailey-Hailey disease. In this work, we used the *Caenorhabditis elegans* model system to gain insight into the downstream response elicited by the loss of *pmr-1* gene. We found that *pmr-1* knocked down animals not only showed defects in the oligosaccharide structure of glycoproteins at the cell surface but also were characterized by reduced susceptibility to bacterial infection. Although increased resistance to the infection might be related to lack of regular recognition of *C. elegans* surface glycoproteins by microbial agents, we provide genetic evidence that *pmr-1* interfered nematodes mounted a stronger innate immune response to Gram-positive bacterial infection. Thus, our observations indicate *pmr-1* as a candidate gene implicated in mediating the worm’s innate immune response.

## Introduction

*Pmr-1* gene encodes a P-type ATPase that regulates Ca^2+^ and Mn^2+^ homeostasis. PMR-1 is located in the Golgi apparatus and it is involved in glycosylation, protein sorting and ER-associated protein degradation in the secretory pathway as demonstrated by studies on the homologous protein of *Saccharomyces cerevisiae* and *Kluyveromyces lactis* yeasts [1–4]. Indeed, a normal concentration of the Ca^2+^ ion inside the Golgi apparatus and the Endoplasmic Reticulum seems to be necessary for the functional activity of the enzymes involved in N- and O-glycosylation, as well as for a regular secretion of glycoproteins and a normal degradation associated with the ER of misfolded proteins [5]. In human, mutations on the PMR-1 orthologues ATP2C1 cause Hailey-Hailey-disease (HHD) [6]. HHD is an autosomal dominant skin disorder in which despite the association of mutation of ATP2C1 with the disease, its *in vivo* role remains poorly investigated [7–9]. The yeast model has provided important information that can explain how in Hailey-Hailey disease loss of ATP2C1 affects human keratinocytes homeostasis [10]. In yeast, inactivation of *pmr-1* leads to multiple alterations, compatible with the observed defects in Ca^2+^ and Mn^2+^ homeostasis. However, the dysregulation in Ca^2+^/Mn^2+^ signaling is supposed to elicit a plethora of downstream alterations that are posited to have different roles in both the yeast phenotype and in the Hailey-Hailey pathogenesis [11]. Similarly, to yeast, in *C. elegans* animal model, PMR-1 is involved in the regulation of Ca^2+^ and Mn^2+^ handling and plays an essential role during its embryonic development [12,13].

In this work, the anti-pathogen response of nematode in combination with specific mutant worms was exploited as an opportunity at identifying the signaling pathways associated with altered *pmr-1* function. Indeed, the anti-microbial defense mechanism of *C. elegans* involves pathways that, although do not appear to be primarily engaged with microbial infection, can dramatically alter pathogen resistance [14,15]. For example, glycosylation is a key modification of proteins and lipids and is involved in various important intermolecular interactions, such as cell signal transduction, differentiation and adhesion, leading to the formation of glycan bindings that are essential for cell viability, communication and developmental processes [16]. In mammalian gastrointestinal tract, external stimuli can modify the expression of glycosyltranferase enzymes, altering the level of glycosylation and causing either an increase or a reduction of the barrier function against pathogens [17]. In the gastrointestinal mucus gel, glycosylated proteins can be membrane-bound, creating a glycocalyx, or secreted, sharing an important protection function [18]. Pathogen infection usually involves host cells adhesion, colonization and, in some cases, invasion of tissues. During pathogen colonization, bacteria normally interact with glycan structures of the host glycocalyx [19]. This interaction determines that the glycosylation state of both host and pathogen changes in response to the presence of the other. Thus, the glycosylation state of the host critically determines protection against pathogens [20–24]. In addition, to cope with host adhesion, pathogen response in *C. elegans* is closely associated with stress, which evokes highly conserved MAP kinases, involved in aging, DNA damage and oxidative stress [25].

In this work, to gain mechanistic insight into the downstream changes due to PMR-1 defects, the impact of *pmr-1* gene knockdown in the glycosylation process and in the response to pathogens was evaluated, using the nematode *C. elegans* as an *in vivo* model system.

## Material and methods

### Bacterial and fungal strains and growth conditions

Gram-negative bacteria strains used in experiments were *Escherichia coli* OP50, *E. coli* ETEC K88 and *Pseudomonas aeruginosa* ATCC 15692; Gram-positive bacteria were *Staphylococcus aureus* ATCC 25923 and *Enterococcus faecalis* JH 2.2. All the strains were grown in Luria Bertani (LB) broth at 37°C overnight, in agitation. *Candida albicans* AT CC 10231 was tested in experiments of fungal infection and was grown in Yeast Extract-Peptone-Dextrose (YPD) broth at 28°C overnight, under shaking. After overnight growth, 30 μl of each culture (about 1 × 10^8^ cells/mL) was spread onto respective agar-plates. For *S. aureus* ATCC 25923 and *C. albicans* ATCC 10231, Trypticase soy agar (TSA) and Brain Heart Infusion (BHI) agar were used, respectively. *E. coli* strains, *E. faecalis* JH 2.2 and *P. aeruginosa* ATCC 15692 were spread on 3.5 cm diameter NGM plates.

### *C. elegans* strains, RNA interference and infection assay

Wild-type *C. elegans* N2 strain, *skn-1*(QV225), *pmk-1*(KU25), *sek-1*(AU1), *hsf-1*(PS3551) and *bus-4(e2693)*CB5443 mutant strains and transgenic SOD-3::GFP CF1553 strain were grown on nematode-growth media (NGM) plates seeded with *E. coli* OP50 at 16°C according to standard procedures [26].

For RNA interference experiments with *pmr-1*, young adult hermaphrodites were fed with HT115 *E. coli* bacteria producing a *pmr-1* dsRNA construct mv ZK256.1c, as described previously [27]. RNAi feeding *E. coli* clones were grown overnight in LB medium containing 100 µg/ml ampicillin and 12,5 µg/ml tetracycline. Worms were grown on RNAi bacteria for 48 h. The empty vector L4440 *E. coli* HT115 strain was used as the negative control. A further control was represented by uninfected N2, grown at 25°C, feeding *E. coli* OP50. After 48 h of RNA interference, animals were transferred onto infection plates, as described above, and grown at 25°C. Animals were monitored daily and scored as dead when they no longer responded to gentle prodding with a platinum wire.

### Estimation of bacterial CFU within the nematode gut

For infection experiments, 10 animals for each sample, after 48 h from infection, were washed and lysed according to [28]. Whole worm lysates were plated onto LB-agar plates. The number of CFU was counted after 24 h of incubation at 37°C, aerobically.

### Rt-qPCR analysis

After 48 h from infection, total RNA from 200 worms for each sample was isolated with RNeasy midi kit (Qiagen) according to manufacturer’s instructions and then digested with 2U/μL DNAse I (Ambion). One microgram of each sample was reverse-transcribed using oligo-dT and enhanced Avian reverse transcriptase (SIGMA, Cat. Number A4464). For RT-qPCR assay, each well contained 2 μL of cDNA used as template, SensiMix SYBR & Fluorescein Kit purchased from Bioline, and the selective primers (200 nM) designed with Primer3 software and reported in Table 1. All samples were run in triplicate. Rotor-Gene Q Real-Time (QIAGEN) was used for the analysis. The RT-qPCR conditions are described by [29]. Quantification was performed using a comparative CT method (CT = threshold cycle value). Briefly, the differences between the mean CT value of each sample and the CT value of the housekeeping gene (*act-1*) were calculated: ΔCT^sample^ = CT^sample^ − CT*^act^*^−1^. Final result was determined as 2^−ΔΔCT^ where ^ΔΔCT^ = ^ΔCTsample^ − ^ΔCTcontrol^.

**Table 1.** Primers for RT-qPCR analysis.

*hsf-1*	FOR	5ʹ-ATGACTCCACTGTCCCAAGG
	REV	5ʹ-TCTTGCCGATTGCTTTCTCT
*pmk-1*	FOR	5ʹ-AAATGACTCGCCGTGATTTC
	REV	5ʹ-CATCGTGATAAGCAGCCAGA
*sek-1*	FOR	5ʹ-CAGAGCCGTTTATTGGGAAA
	REV	5ʹ-TGCATCCGGCTTGTACAGT
*sod-3*	FOR	5ʹ-AGAACCTTCAAAGGAGCTGATG
	REV	5ʹ-CCGCAATAGTGATGTCAGAAAG
*act-1*	FOR	5ʹ-GAGCGTGGTTACTCTTTCA
	REV	5ʹ-CAGAGCTTCTCCTTGATGTC
*gly-11*	FOR	5ʹ-GGACCTGCGGTGGAGAACT
	REV	5ʹ-GCGGAAAATGTGGCCAACT
*gmd-2*	FOR	5ʹ-AAAGCGAGCTGACCCCATT
	REV	5ʹ-ATACATCTTGGCGACCGCATA
*let-653*	FOR	5ʹ-CTGTCTCGTGAGAATATGTCC
	REV	5ʹ-TTCCACGTCGTCGCATGT
*osm-8*	FOR	5ʹ-AGAAGCCCCACCACTGATTG
	REV	5ʹ-TTGTTTTTGCCACGGTTCAA
*act-1*	FOR	5ʹ-GAGCGTGGTTACTCTTTCA
	REV	5ʹ-CAGAGCTTCTCCTTGATGTC

### Analysis of *C. elegans* strain SOD-3::GFP fluorescence

Synchronized transgenic worms were transferred on RNAi NGM plates and then infected, as described above. After 48 h from RNAi and 24 h or 48 h from infection, worms were anesthetized and observed as described in [30].

### Measurement of reactive oxygen species (ROS)

ROS formation in *C. elegans* was measured using the fluorescent probe H_2_DCFDA according to [31].

### Lectin staining

Lectin staining of live nematodes after 48 h from infection was performed as described previously [32]. After fixation, 40 μL of stained worms were mounted on glass slides and observed under a Zeiss Axiovert 25 microscope. Texas Red conjugated *Agaricus bisporus lectin* (ABA) and *Anguilla Anguilla lectin* (AAA) and FITC conjugated *Ulex europeus lectin* (UEA), *Canavalia ensiformis* (Con-A), *Galanthus nivalis lectin* (GNA), were used in this study. ABA binds Gal(β1,3)GalNAc, UEA recognizes α-L-Fucose, Con-A is specific for Man(α1,6)Man(α1,3)Man, GNA recognizes Man(α1,3)Man, AAA binds L-Fuc(α1,2)Gal. To assess the fluorescent signal of the AAA, ConA and ABA lectins, worms were analyzed with an Axio Observer inverted microscope equipped with the ApoTome System (Carl Zeiss Inc.), in a series of 1.0 µm sequential sections and processed with the AxioVision software (Zeiss). Digital images were obtained from the 2D reconstruction of selected serial optical sections.

### TEM analysis

For trasmission electron microscopy (TEM), nematodes were processed as previously described with some minor differences [33]. Briefly, samples were fixed in 2% glutaraldehyde in PBS for 24 h at 4°C, post-fixed in 1% OsO4 for 2 h, stained for 1 h in 1% aqueous uranyl acetate, and pre-embedded in a thin 10% gelatin gel overnight. Gelatin small blocks were cut to have worms close to each other. Small pieces were dehydrated with graded acetones and embedded in Epon-812 (Electron Microscopy Sciences). Semithin sections stained with 1% methylene blue were used to select suitable areas of ultrastructural sectioning. Uranyl acetate/lead citrate-stained ultrathin sections were examined with a Philips CM10 and Morgagni 268D transmission electron microscope (TEM; FEI- Italia srl; Termofisher Scientific).

### Statistical analysis

The statistical significance was performed by Student’s t-test or one-way ANOVA analysis coupled with a Bonferroni post test (GraphPad Prism 5.0 software, GraphPad Software Inc., La Jolla, CA, USA). Differences with p values < 0.05 were considered significant and were indicated as follows: *p < 0.05, **p < 0.01, and ***p < 0.001. Experiments were performed at least in triplicate. Data were presented as mean ± SD.

## Results

### *Pmr-1* silencing altered glycoconjugates and enzymes expression in *C. elegans*

In order to evaluate the effect of the inactivation of the Ca^2+^-ATPase PMR-1 in *C. elegans* on glycosylation process, the *pmr-1* gene was silenced by feeding RNAi. RT-qPCR analysis demonstrated that, after 48 h of treatment, the gene silencing occurred. Indeed, the levels of *pmr-1* transcript of individuals subjected to RNA interference, normalized for the housekeeping gene *act-1*, were considerably lower (about 70%) than the transcript derived from nematodes treated with the empty vector (Fig. S1). Different lectins were used to characterize cellular surfaces and surface coat of wild-type nematodes after RNAi treatment.

**Figure 1.**
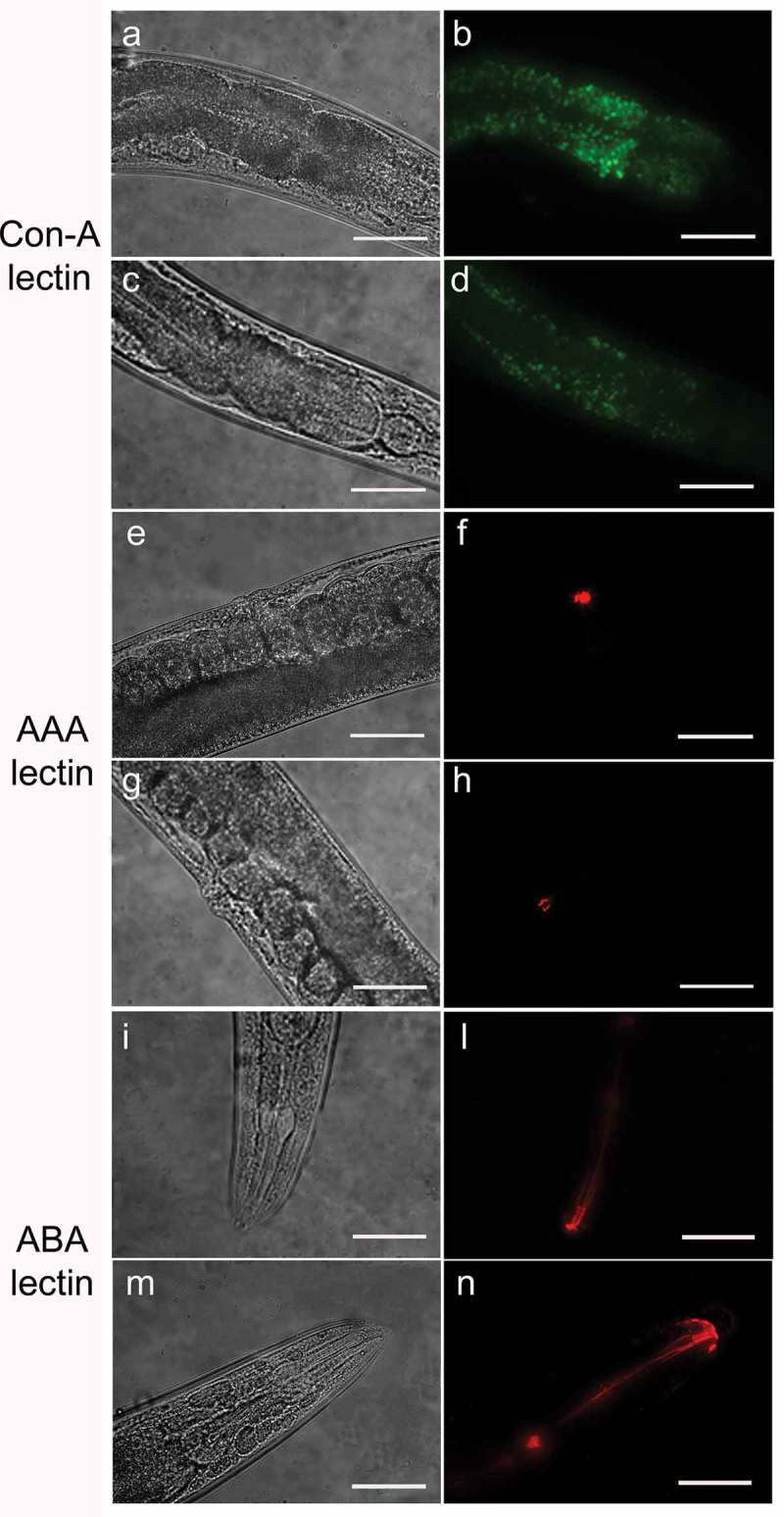
Lectin staining of N2 e *pmr-1* mutant worms. Nematodes were stained with FITC conjugated Con-A (a, b, c and d), Texas red conjugated AAA (e, f, g and h) or ABA (i, l, m and n) lectins after RNAi. Panels a, b, e, f, i and l indicate worms interfered with the empty vector. Panels c, d, g, h, m and n indicate *pmr-1* mutant worms. *n*= 10 for each sample. Scale bar = 100 μm.

The use of lectins allowed us to evaluate the presence of alterations of glycoproteins on *C. elegans* mutant surfaces. With Con-A lectin staining, the fluorescence was considerably less intense throughout the body of the nematode in *pmr-1* worms with respect to control (Figure 1). The ABA lectin labeling was more evident in the region of the pharynx and gut of *pmr-1* nematodes, compared to control nematodes. Moreover, an altered fluorescent signal was also observed in vulva of *pmr-1* nematodes, when AAA staining was carried out (Figure 1). On the contrary, no changes were observed when nematodes were treated with UEA or GNA lectin staining (Fig. S2). These data indicate that silencing *pmr-1* gene expression dramatically alters *C. elegans* glycoproteins abundance and distribution.

**Figure 2.**
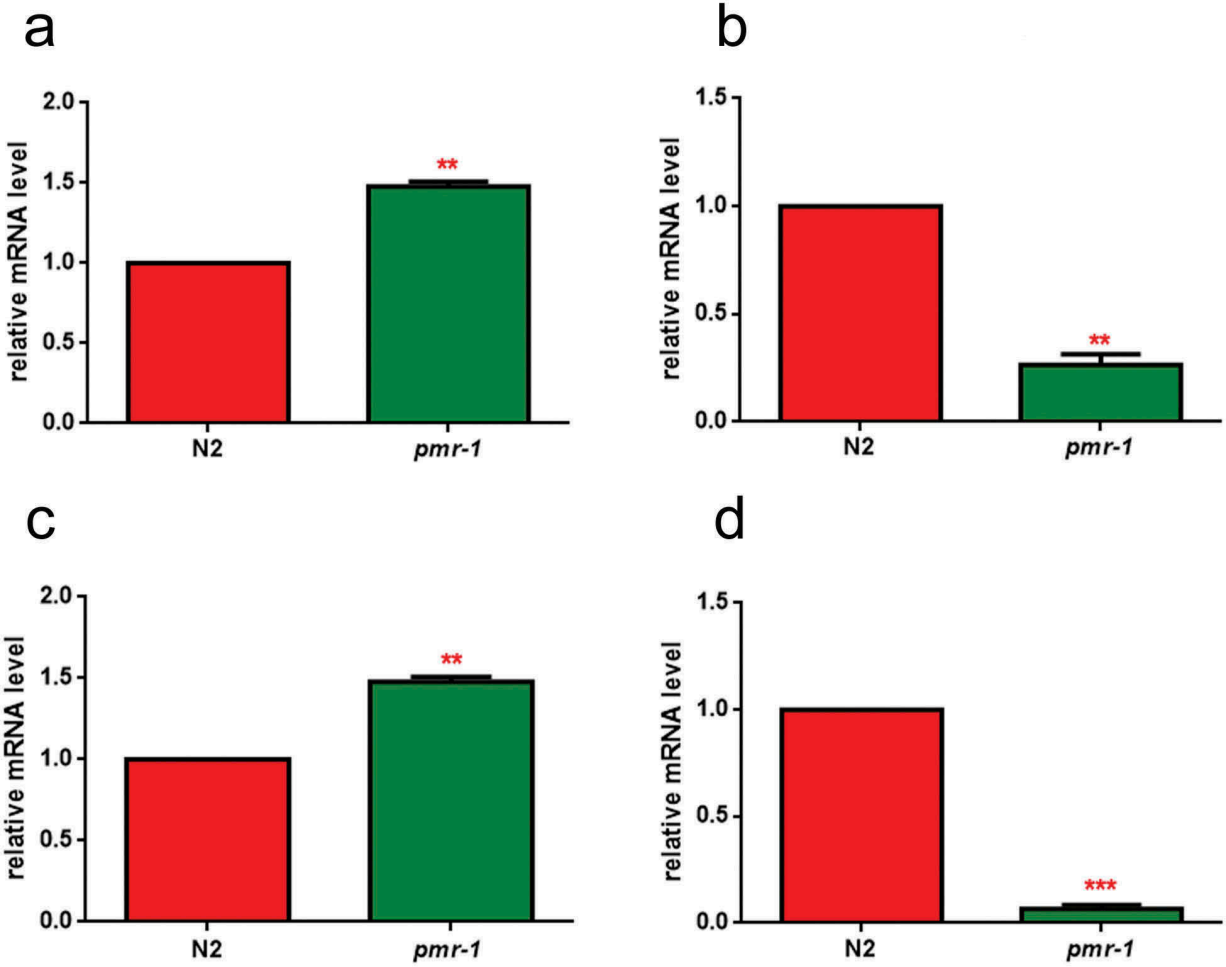
RT-qPCR analysis of glycosylation genes. Expression of (a) *gly-11*, (b) *gmd-2*, (c) *let-653* and (d) *osm-8* genes in N2 worms and *pmr-1* mutants. Histograms show the expression of glycosylation-related genes detected by real-time PCR. Bars represent the mean of three independent experiments. Asterisks indicate significant differences (**p < 0.01, ***p < 0.001).

In order to identify possible alterations in gene expression involved in glycosylation processes, real-time qPCR analysis was performed for the N-acetylgalactosaminyltransferase *gly-11*, the GDP-mannose 4,6-dehydratase *gmd-2* and mucin-like *let-653* and *osm-8* transcripts. The results showed that *pmr-1* silencing induced an increased transcription of *gly-11* and *let-653*, and a reduction in the mRNA levels for *gmd-2* and *osm-8*, further supporting the involvement of *pmr-1* gene in glycoconjugates formation (Figure 2).

### *Pmr-1* suppression in *C. elegans* induced resistance to staphylococcus aureus infection

**Figure 3.**
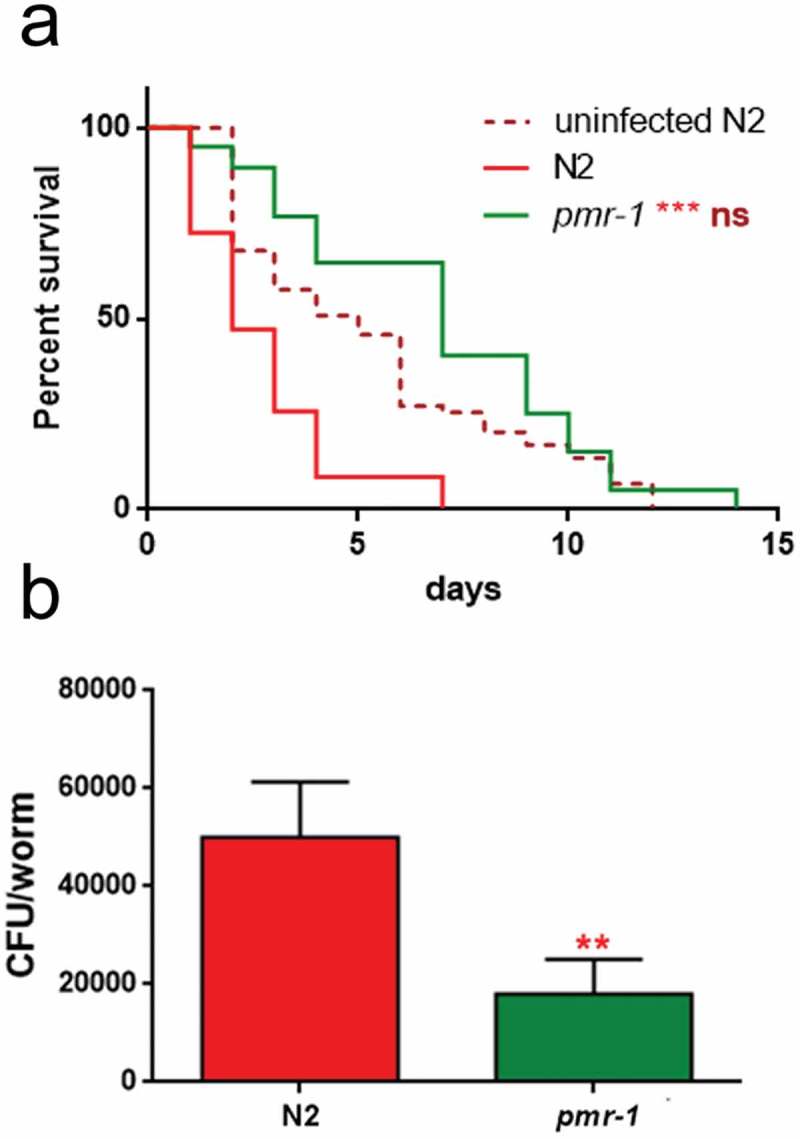
Effect of *pmr-1* silencing on *C. elegans* viability and gut colonization. (a) Kaplan-Meier survival plot of *pmr-1* mutant worms with respect to controls (infected or uninfected N2). *n* = 60 for each data point of single experiments. (b) Bacterial colony-forming units (CFU) recovered from nematodes after 48 h to infection with *S. aureus*. Bars represent the mean of three independent experiments. Asterisks indicate significant differences (**p < 0.01, ***p < 0.001).

**Figure 4.**
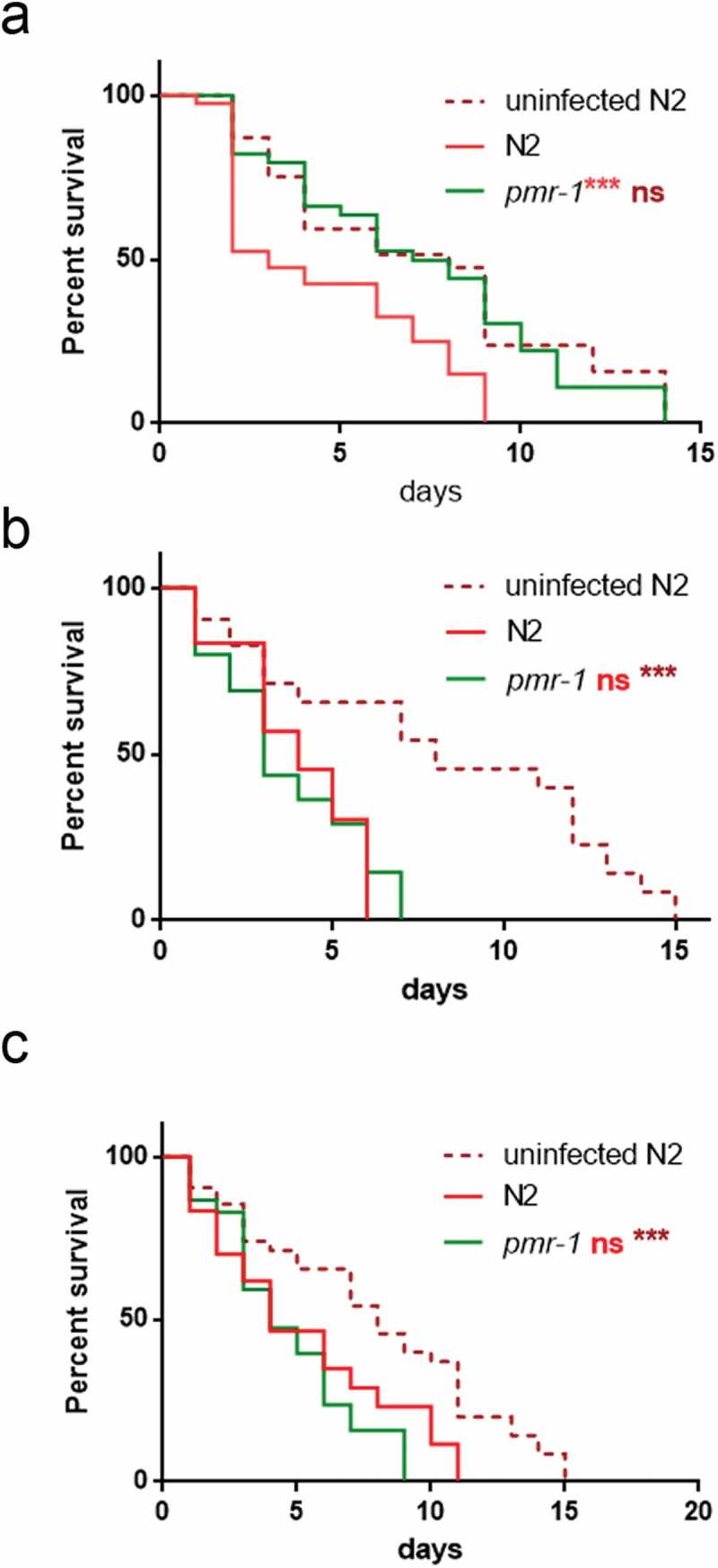
Effect of *pmr-1* silencing on worms infected with different pathogens. Kaplan-Meier survival plot of N2, uninfected wild-type and *pmr-1* mutant worms infected with (a) *E. faecalis*, (b) *P. aeruginosa* and (c) *C. albicans. n*= 60 for each data point of single experiments. Asterisks indicate significant differences (***p < 0.001, ns: not significant).

Glycoconjugates have been implicated in the modulation of the nematode response to infection. Since *pmr-1* mutants showed an altered glycoconjugates pattern, we evaluated the effect of *pmr-1* silencing on bacterial infections. Toward this aim, RNAi interfered nematodes were exposed to the Gram-positive pathogen *Staphylococcus aureus* and its effects on worm physiology were evaluated. We found that *pmr-1* nematodes exhibited an increased lifespan when compared to wild-type animals, similarly to uninfected worms. In particular, the median survival was of 7 days in the *pmr-1* worms, 2 days in the infected control nematodes and 5 days in uninfected N2 worms (Figure 3a). *C. elegans* lifespan has been linked to the control of intestinal bacterial accumulation [34]. Additionally, bacterial colonization and proliferation in the worm’s intestine is considered an important contributor to bacterial virulence [35]. To investigate the relationship between *S. aureus* load and the *pmr-1* worms increased longevity, we measured the numbers of viable bacteria (Colony Forming Units, CFU) recovered from infected worms. In the control worms grown in the presence of *S. aureus* by day 2, when the >50% have died, the intestinal load was >5 × 10^4^ CFU/worm. In contrast, *pmr-1* worms had about 60% lower colonization by *S. aureus* (Figure 3b) consistent with their increased lifespans. Furthermore, to assess the significance of our observations, we investigated how different bacterial species affect colonization and lifespan of *pmr-1* defective worms. Thus, *pmr-1* worms were exposed to either Gram-positive pathogen *Enterococcus faecalis* or Gram-negative bacteria *Pseudomonas aeruginosa*. We found that lifespan of *pmr-1* defective worms exposed to *E. faecalis* was higher than the wild-type controls resembling the response of *pmr-1* worms exposed to *S. aureus* (Figure 4a). In particular, the median survival was of about 8 days in the *pmr-1* worms and 3 days in the infected control nematodes. In contrast, we found that the response to *P. aeruginosa* did not show significant differences between mutant and infected control population (Figure 4b).

Additionally, evaluation of *C. elegans* survival in response to either *Candida albicans or* Gram-negative *Escherichia coli* ETEC K88 exposure showed a similar lifespan between *pmr-1* and infected control worms (Figure 4c and data not shown, respectively).

These observations indicate that defective *pmr-1* function selectively influences signaling pathways that are specific to Gram-positive bacterial infection.

### *Pmr-1* mutant worms showed microvilli surrounded by a dense glycocalyx

**Figure 5.**
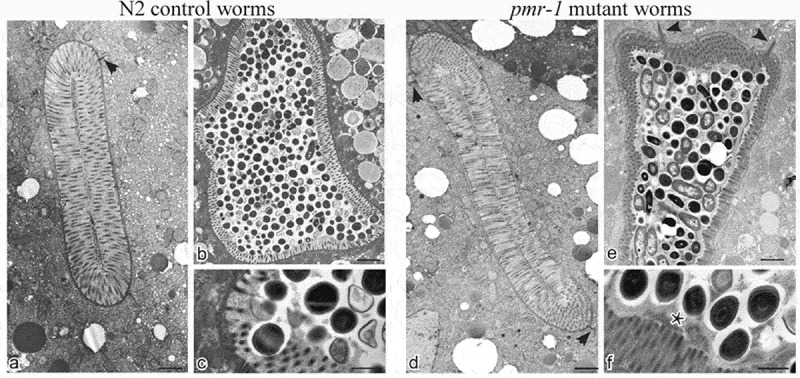
Ultrastructural analysis of control worms and *pmr-1* mutants, before and after infection with *S. aureus.* Electron micrographs show the intestinal surface of both control and *pmr-1* nematodes with a typical apical domain, including brush border with microvilli, terminal web, and apical junctions (a, d; arrowheads). Panels a and d indicate N2 and *pmr-1* worms before *S. aureus* infection, respectively. Panels b and c indicate control worms after *S. aureus* infection, while *pmr-1* infected worms were indicated in panels e and f. After infection (b, e), the intestinal lumena of both control and defective worms are dilated. However, in mutant worms, a denser and thicker glycocalyx (asterisc) separates the microvilli from the intralumenal bacteria (e-f). Differently, in control worms, bacteria adhere to the microvilli (b, c). Bars 1 µm; Fig. c, f. Bars 0,5 µm.

In the intestinal tract, carbohydrate–protein interactions have important roles in the distinct aspect of the host–microbe interaction. These interactions regulate host–microbe exchange of nutrient with a mutual benefit to both organisms [17]. These interactions are also involved in both pathogen recognition and in the cellular interaction that lead to pathogen neutralization [36]. Additionally, microbe–host interaction alters the worm intestinal structure that might either facilitate or inhibit pathogen recognition and neutralization [37]. Thus, to evaluate possible alterations in the intestinal lumen of *pmr-1* worms and to gain insight into the mechanisms involved in its resistance to *S. aureus* infection, we imaged *C. elegans* using transmission electron microscopy (TEM). In 3-days adults, by TEM analysis the intestinal morphology appears uniform in its size and shape in both *pmr-1* and control worms. Cross and longitudinal sections revealed intestinal cells linked by adherens junctions at their apical borders. They form a narrow lumen bordered by dense microvilli and coated along the entire luminal surface by a dense glycocalyx (Figure 5a,d) which functions to protect microvilli from physical or toxic injury. These also serve for enzyme digestive localization and filtration of absorbed components [38].

After *S. aureus* infection, bacteria were visible in the intestinal lumen of both *pmr-1* and control worms (Figure 5b,e). However, in control animals, bacteria accumulated in the intestinal lumen that becomes dilated, displaying shorter microvilli relative to the infected worms (Figure 5b). Additionally, in control nematodes dark staining individual bacteria strictly adhered to or were even embedded into microvilli (Figure 5b,c), suggesting that they might be responsible for local damage via degradation of the microvilli, a process already described during aging (Figure 5b,c). Differently, the intestinal apical surface of *pmr-1* worms showed the typical dense brush border constituted of microvilli surrounded by a dense glycocalyx (Figure 5f). This is consistent with a protective role of the glycoproteins composing the glycocalyx that, despite the persistent bacterial infection, might maintain the intestinal lumen integrity and appear to be protected from bacterial invasion.

### *Pmr-1* influenced oxidative stress response before and during infection

**Figure 6.**
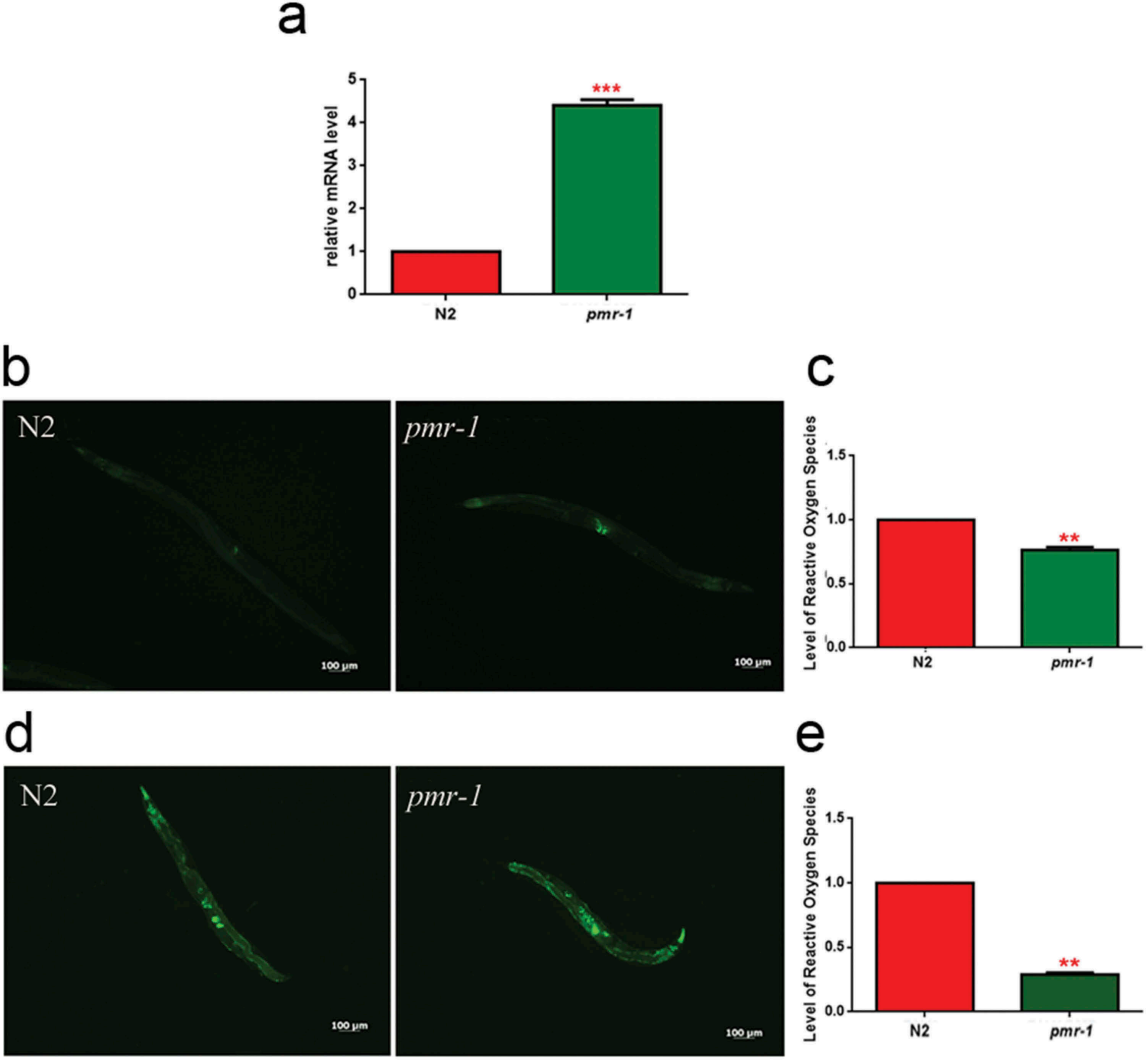
Analysis of oxidative stress in SOD-3::GFP strain and ROS evaluation. (a) Expression of *sod-3* mRNA in N2 and *pmr-1* worms. (b) Fluorescence microscopy of SOD-3::GFP worm strain. Scale bar = 100μm. (c) Measurement of ROS levels in N2 worms and *pmr-1* mutants. (d) Fluorescence microscopy of SOD-3::GFP worm strain after 48 h of infection with *S. aureus*. Scale bar = 100μm. (e) Measurement of ROS levels in N2 and *pmr-1* worms after 48 h of RNAi. Statistical analysis was evaluated by one-way ANOVA with the Bonferroni posttest; asterisks indicate significant differences (**p < 0.01; ***p < 0.01). Bars represent the mean of three independent experiments.

**Figure 7.**
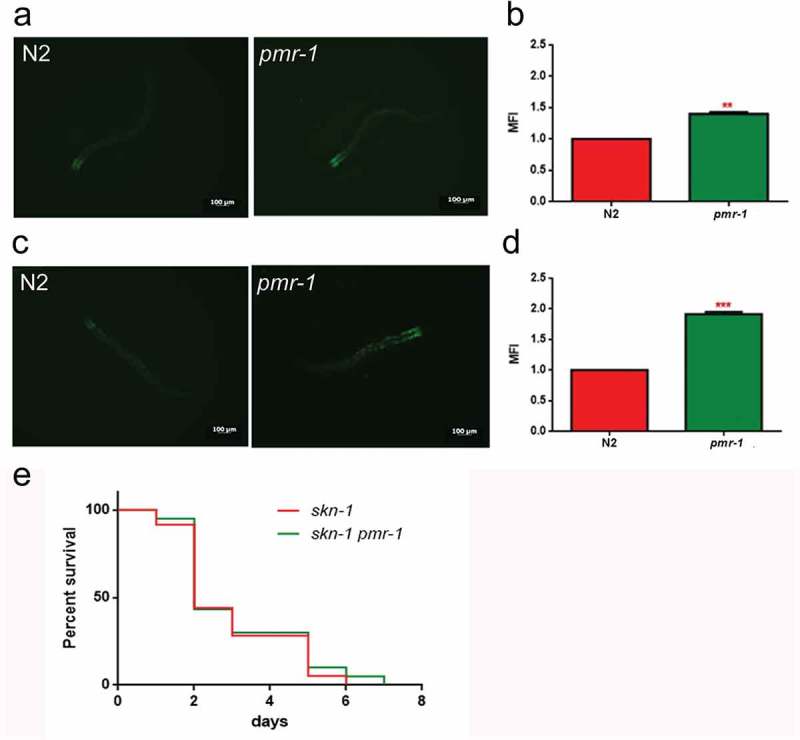
Analysis of oxidative stress in SKN-1::GFP strain. (a) Fluorescence microscopy of SKN-1::GFP worm strain after 48 h of RNAi and (b) related median fluorescence intensity. (c) Fluorescence microscopy of SKN-1::GFP worm strain after RNAi and S. aureus infection and (d) related median fluorescence intensity. Statistical analysis was evaluated by one-way ANOVA with the Bonferroni posttest; asterisks indicate significant differences (**p < 0.01; ***p < 0.01). Bars represent the mean of three independent experiments. Scale bar = 100μm. (e) Kaplan-Meier survival plot of *skn-1* mutant worms with silenced *pmr-1*, after 48 h to infection with *S. aureus*, as compared to control. (ns: not significant). *n* = 60 for each data point of single experiments.

In worms exposed to bacterial infection, an antioxidant stress response is required for the survival of worms, since it provides protection against oxidative stress produced by pathogenic bacteria [39]. In light of the extended lifespan of nematodes infected with *S. aureus*, we hypothesized that oxidative stress response genes will be upregulated in *pmr-1* worms. The *sod-3* gene in *C. elegans* encodes the mitochondrial MnSOD isoform 3 of superoxide dismutase. In nematodes, as in mammals, the SOD family enzymes represent the first line of defense against oxidative stress, contributing to neutralize reactive oxygen species (ROS) through the reaction that transforms superoxide ion into hydrogen peroxide and molecular oxygen. Then, hydrogen peroxide can be neutralized by catalase or glutathione peroxidase, but SOD proteins are the only ones able to neutralize the superoxide ion [40]. Thus, expression of *sod-3* gene was examined using quantitative real-time PCR in control and *pmr-1* worms. The expression of *sod-3* gene was significantly higher in the *pmr-1* strain compared to control (Figure 6a). Using a SOD-3::GFP transgenic strain as a read-out of both SOD-3 protein expression and of antioxidant stress response activation, we observed a strong induction of the SOD-3::GFP reporter in *pmr-1* worms (Figure 6b). Consistently, endogenous ROS level was also decreased upon *pmr-1* treatment (Figure 6c). As shown in Figure 6d, fluorescence signal of SOD-3 protein was also increased in response to pathogen exposure both in *pmr-1* and in control worms. Interestingly, when fed with *S. aureus*, ROS levels were robustly reduced in *pmr-1* nematodes compared to control (Figure 6e). The reduced oxidative stress levels were also consistent with the higher fluorescence signal of SOD-3 protein (Figure 6e). In *C. elegans*, SKN-1, the orthologue of the mammals NRF2, mainly control the transcription of several enzymes that sustain the detoxification reactions in response to ROS. Consistently, SKN-1 is required for the survival of the worms and provides protection against pathogenic bacteria [41]. Therefore, we investigated whether the extended lifespan of the *pmr-1* worms fed *S. aureus* was associated with the expression of *skn-1* gene. We observed that SKN-1::GFP transgenic *pmr-1* nematodes constitutively express SKN-1 protein with a higher fluorescence detected at the level of intestinal cell nuclei, as compared to control (Figure 7a,b). Additionally, SNK-1 expression resulted in significantly increased in the *pmr-1* worms compared to the control worms after *S. aureus* infection (Figure 7c,d). The involvement of SKN-1 was also demonstrated in lifespan experiment, where *skn-1/pmr-1* mutant strain exhibited increased susceptibility to *S. aureus* similarly to control animals (Figure 7e).

Therefore, collectively these observations indicate that the life-extending effect of *pmr-1* inhibition might be mediated through activation of an antioxidant response.

### *PMK-1* gene was involved in innate immunity responses mediated by *PMR-1*

**Figure 8.**
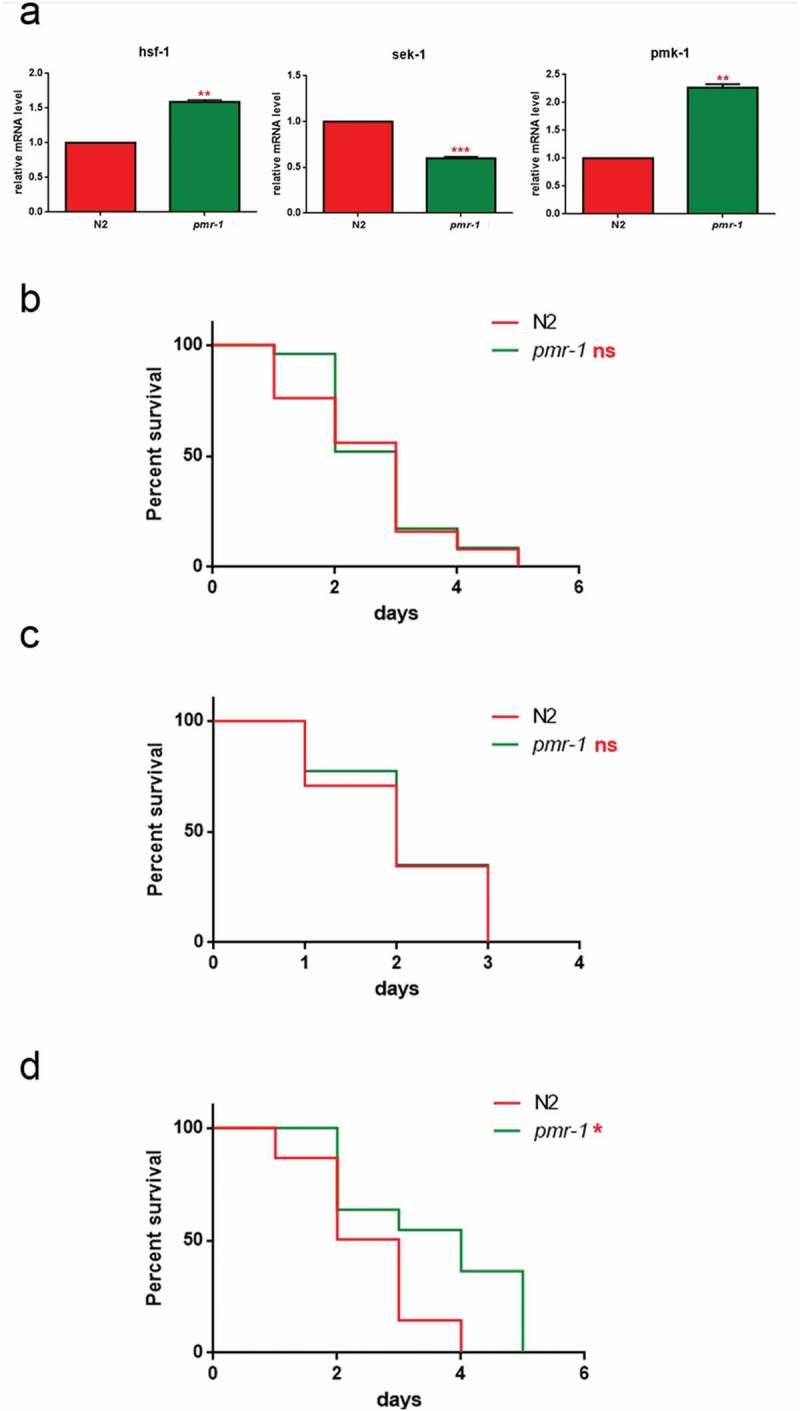
Impact of *pmr-1* silencing on *C. elegans* immunity. (a) Expression of *hsf-1, sek-1* and *pmk-1* mRNA level in *pmr-1* worms, as compared to control. Bars represent the mean of three independent experiments. Kaplan-Meier survival plot of (b) *pmk-1* (c) *sek-1* and (d) *hsf-1* mutant worms with silenced *pmr-1*, after 48 h to infection with *S. aureus*, as compared to control. Asterisks indicate significant differences (*p < 0.05, **p < 0.01, ***p < 0.001, ns: not significant). *n* = 60 for each data point of single experiments.

In response to infection by pathogens, ROS generation plays a key role in worm innate immunity. However, aberrant upregulation of ROS production can also be detrimental to the host. Thus, ROS levels must be tightly regulated to be efficiently utilized by the host-defense response. In this context, the p38 MAPK family member 1 (PMK-1) appears to be the most commonly evoked factor in the worm’s pathogen defense. Loss of *pmk-1* not only had a significant negative effect on pathogen resistance but also negatively contribute to worm lifespan [42]. In this dual activity of *pmk-1*, two parallel downstream pathways work to mediate its effects. *Pmk-1* regulates both the transcriptional activation of the majority of innate immune response genes through ATF-7 and the activation of the antioxidant response through SKN-1. Thus, to investigate the mechanism of the increased lifespan of *pmr-1* worms in response to *S. aureus* infection, transcriptional levels of *pmk-1* and the related SAPK/ERK (*sek-1)* [43,44] were evaluated. In *pmr-1* worms, an increased expression of *pmk-1* transcripts was observed as compared to control (Figure 8a). Conversely, *sek-1* transcripts resulted in reduced in *pmr-1* nematodes compared to control animals.

To determine the overall contribution of both PMK-1 and SEK-1 kinases on worm defense to *S. aureus*, we measured susceptibility to infection in animals lacking expression of either *pmk-1* or *sek-1* after RNAi-mediated knockdown of *pmr-1*. RNAi-mediated knockdown of *pmr-1* in *pmk-1* mutant strain exhibited reduced susceptibility to *S. aureus* similarly to the wild-type animals treated with RNAi-*pmr-1* alone (Figure 8b). Conversely, RNAi-mediated knockdown of *pmr-1* in *sek-1* mutant strain exhibited enhanced resistance to the *S. aureus* infection (Figure 8c). An important requisite of the pathogen defense response is the activation of the endoplasmic reticulum unfolded protein response (UPR/ER) to ensure tolerance and survival to the infection [45]. The protein folding stress response system is regulated by the transcription factor HSF-1, whose inactivation has been shown to render worms sensitive to infection [46]. To test whether HSF-1 is involved in the protection of *S. aureus* infection observed in *pmr-1* animals, we analyzed the effect of *hsf-1* mutation in this context. As shown in Figure 8c, *pmr-1* animals were characterized by increased *hsf-1* expression. Consistently, *pmr-1* treatment in *hsf-1* mutant strain was susceptible to *S. aureus*, similarly to the control animals (Figure 8d). These results indicate that *pmr-1* inactivation leads to protection by eliciting multiple antimicrobial defense pathways.

### *PMR-1* silencing enhanced resistance in *C. elegans Bus-4* mutants

**Figure 9.**
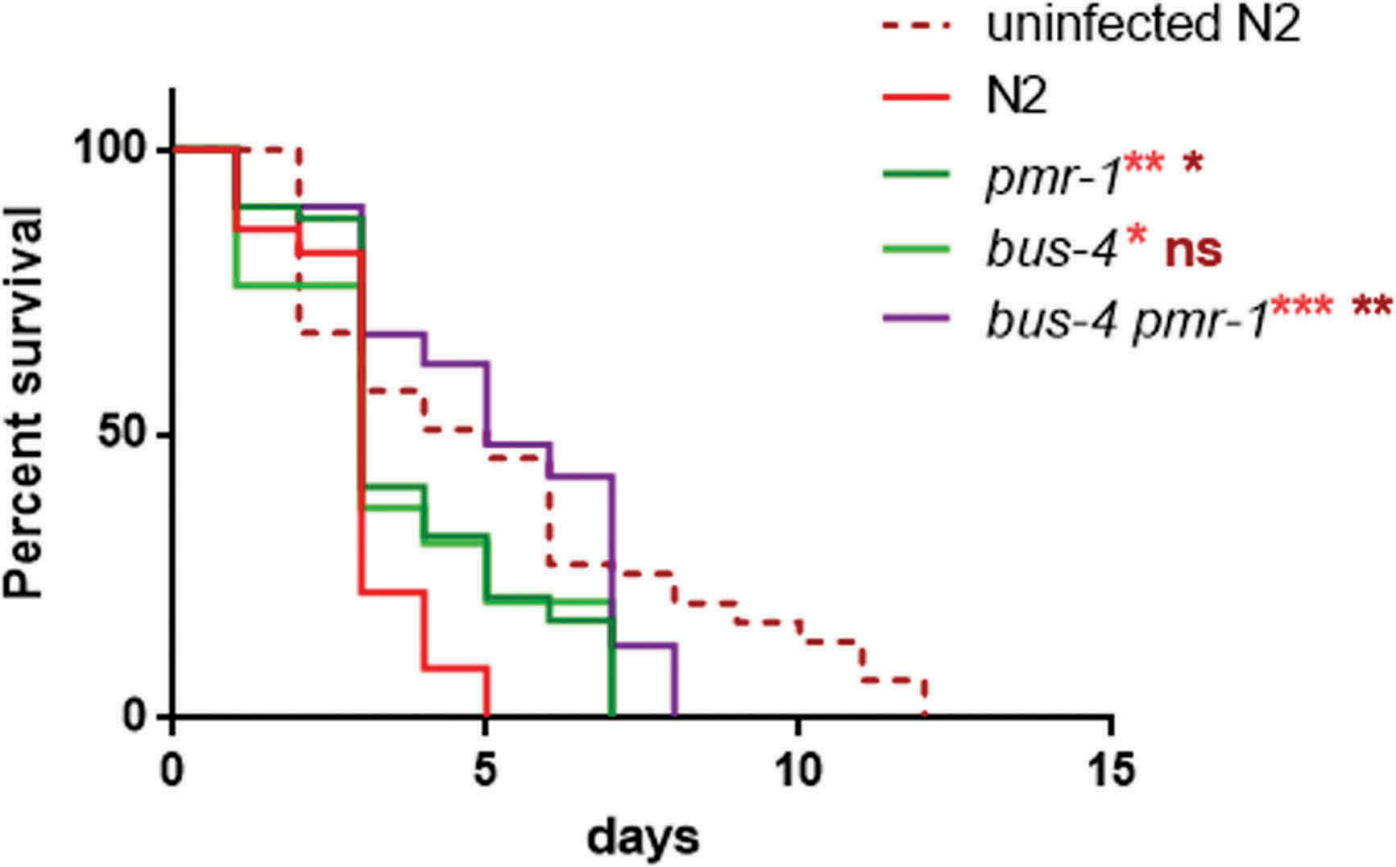
Lifespan analysis. Kaplan-Meier survival plot of infected and uninfected N2 and *bus-4* worms, after 48 h of RNAi, infected with *S. aureus. n* = 60 for each data point of single experiments. Asterisks indicate significant differences (*p < 0.05; **p < 0.01, ***p < 0.001).

It has been proposed that pathogens bind to a surface-exposed glycan epitope and worm mutants in either O- and N-glycosylation are resistant to infection due to the failure of pathogens to bind to the host surface [47,48]. According to our observations, it seems likely that *pmr-1* inactivation leads to protection against infection by eliciting multiple antimicrobial defense pathways. However, it is possible that a PMR-1-dependent signaling is required for the production or surface exposure of a glycan epitope. In yeast, we and others have shown a dual role of *pmr-1* in the secretory pathway and in N and O-linked glycosylation. Thus, we investigate whether the increased lifespan in *pmr-1* animals could be linked to the altered surface expression of glycans. Toward this aim, we performed RNAi-mediated knockdown of *pmr-1* in animals with loss of function of *bus-4* which encodes a glycosyltransferase [49]. *Bus-4* mutants are resistant to *S. aureus* infection as they not only have changes in O- and N glycans but also marked changes in the expression of the surface glycan peptides *let-653* and *osm-8* [20]. We found that *bus-4* mutants exhibited reduced susceptibility to *S. aureus* similarly to that of *pmr-1* animals (Figure 9). Interestingly, the double *bus-4*/*pmr-1* mutants were more resistant to *S. aureus* infection than either the single *bus-4* or *pmr-1* mutant worms (Figure 9). These observations are therefore consistent with the notion that *pmr-1* knockdown might act on *S. aureus* resistance only in part because of the glycosylation impairment.

## Discussion

PMR-1 has been functionally characterized in yeast; in human, mutations in the orthologous ATP2C1 cause the skin disease Hailey-Hailey. However, despite the fact that essential functions of PMR-1 depend on its ability to regulate the Ca^2+^ and Mn^2+^ homeostasis, the concept of how *pmr-1* influences cell biology remains largely speculative in both yeast and human. Thus, causative pathway and disease mechanisms have yet to be definitively identified. To address the lack of functional evidence, in this study we applied a classical genetic approach to evaluate how depletion of *pmr-1* gene affects the susceptibility of worms to bacterial infection. Anti-pathogen response of nematode in combination with specific mutant worms was used to identify signaling pathways associated with altered *pmr-1* function. We identified two aspects of host-physiology, glycosylation and antimicrobial responses that in *pmr-1* depleted animals were dysregulated.

A specific labeling of glycans on the surface of *pmr-1* worms showed an altered pattern of the lectin binding compared to the control worms indicating that *pmr-1* depletion leads to an altered glycosylation pattern and surface properties. In particular, we found that the fluorescent signal related to the binding of the AAA lectin to the surface glycans was altered with respect to control. The specificity of this lectin for the L-Fuc disaccharide (α1,2) Gal may relate to a different presence of fucosylated surface glycans in *pmr-1* mutants compared to nematodes with normal transcript levels. Fucose residues in the glycan structures are typical of invertebrate organisms. There are divergent opinions on the location of fucose residues in *C. elegans*: fucose could be linked to terminal residues or one or two fucose residues could substitute the N-acetylglucosamine core linked to asparagine [23]. Therefore, defects in glycosylation process could cause alterations of the central nucleus linked to the Asn residue or the terminal stage of the process in which an abnormal addition of fucose residues occurs. Fucose also substitutes Ce Core II O-glycans, which are increased in abundance in *bus-2* and −4 mutants [20,32]. The ABA lectin is specific to the disaccharide Gal (β1,3) GalNAc, which represents the central nucleus that binds to the Ser/Thr residue in the O-glycans. The signal related to ABA lectin staining was more intense in *pmr-1* nematodes and it was located at the level of the pharynx of worms. This result probably was due to alterations of O-glycans containing this central core, caused by defects in the glycosylation process. The observed alterations in lectin staining correlated to the different expression of genes involved in glycosylation processes, confirming the presence of modifications in glycoconjugates. In particular, *gmd-2* catalyzes the conversion of GDP-Man to GDP-Fuc, involved in the synthesis of fucosyl glycoconjugates [20]. The reduction of transcript levels of *gmd-2* in *pmr-1* mutants were consistent with the weaker fluorescence signal observed in *pmr-1* worms stained with AAA lectin.

Glycoconjugates are involved in the interaction of *C. elegans* with the external environment since they are targets for the adhesion of microorganisms to the cuticle and to the cell surface. Several studies showed the importance of the structure, density and distribution of glycoconjugates in the host–pathogen interaction and in the determination of the susceptibility or resistance of the nematode to bacterial attack [32,49]. (Gravato-Nobre MJ et al., 2011; Palaima et al., 2010). Consistent with an altered glycosylation pattern and surface properties, nematodes subjected to RNAi for *pmr-1* gene showed an increased longevity compared to control individuals, upon infection with *S. aureus*. The increased resistance to infection was related to the low colonization capability of the Gram-positive bacteria observed in *pmr-1* mutants, as compared to control. Intestinal pathogens exploit host immune responses to compete with the indigenous microbiota and therefore to colonize the gut. High colonization capability in *C. elegans* gut, followed by the death of the worms, could permit bacterial survival, and protection by stress so as to promote environmental spread [50].

It has been reported that some glycosylation genes in *C. elegans* determine susceptibility to pathogens. Indeed, the availability of *C. elegans* mutants for genes involved in the glycosylation of extracellular matrix proteins allowed for observation of an increased nematode resistance to the infection of the Gram-positive *Microbacterium nematophilum* and to the formation of biofilm by the Gram-negative *Yersinia pestis* [20,32,47]. In particular, it was observed that *C. elegans* mutants for the glycosyltransferase *bus-4* gene, resistant to the *M. nematophilum*, showed an increase in fucosylated N-glycans and alterations in the central core Gal (β1,3) GalNAc of the O-glycans [20]. Moreover, mutations in eight *squashed vulva* (*sqv*) genes in worms cause defects in cytokinesis during embryogenesis and in vulva morphogenesis during postembryonic development [51]. Mutants in glycosyltransferases *bre* genes are resistant to killing by Cry toxins produced by the Gram-positive *Bacillus thuringiensis* because they are involved in the synthesis of the receptor for these toxins on the intestinal cells surface [52]. Therefore, composition of the glycoproteins on *C. elegans* cell surface is fundamental for the correct recognition by the receptors of Gram-positive bacteria and for the subsequent phases of adhesion and pathogenesis. *S. aureus* produces adhesion molecules to the host cells that include the MSCRAMM family (surface microbial components that recognize matrix adhesive molecules), capsular polysaccharides and teichoic acids [53,54]. In particular, glycosylation of teichoic acids is involved in mechanisms of Gram-positive interaction with the host [55]. Alterations of the glycosylation process, due to the absence of the functional activity of PMR-1 may cause changes in the structure of glycoproteins exposed on the cell surface. These glycoproteins could be implicated in binding to *S. aureus* receptors; hence, their alteration would compromise the adhesion of the bacterium to the nematode, reducing its ability to infect the host. Consistently, we observed an increased resistance of the *pmr-1* mutant nematodes to *S. aureus*. However, the additive phenotype of the double mutant indicates that the two mutated genes function independently from the other by differentially influencing the expression of surface-exposed glycoconjugates involved in bacterial adhesion colonization or alternatively *pmr-1* plays a role in controlling immune response of *C. elegans* to infections. In line with a model in which *pmr-1* is involved in response to the infection besides its involvement in the surface expression of glycoconjugates, we found the central signaling modules of the antimicrobial response of *C. elegans* to be dysregulated by loss of *pmr-1*. We found that upon *pmr-1* knockdown both PMK1/SKN-1 and PMK1/HSF-1 signaling, which mediate stress and immune responses, was activated [1,56]. The extended lifespan in *S. aureus*-fed *pmr-1* worms thus largely depend on the activation of these pathways that contribute to enhance pathogen resistance and longevity, as revealed by the experiments in the *skn-1* mutant.

In summary, we found that *pmr-1* is involved in the surface expression of glycoconjugates in *C. elegans*. Furthermore, we discovered that behind its role in the glycosylation process, *pmr-1* function is involved in the control of immune and oxidative stress homeostasis.

## Aknowledgements

The authors acknowledge the *Caenorhabditis Genetics Center* (University of Minnesota, Minneapolis) for the nematode and *E. coli* OP50 strains.
