# Supplementary material for: Pmr-1 gene affects susceptibility of Caenorhabditis elegans to Staphylococcus aureus infection through glycosylation and stress response pathways' alterations
Source: Virulence. 2019 Nov 27;10(1):1013–25. doi: 10.1080/21505594.2019.1697118 (PMC6930020; doi:10.1080/21505594.2019.1697118)
Supplement: Supplemental Material [file kvir-10-01-1697118-s001.zip › Figure Suppl caption.docx]

**Figure S1. RT-qPCR analysis of *pmr-1* gene.** Expression of *pmr-1* mRNA level in N2 worms after 48 h of RNAi, as compared to control interfered with empty vector. Bars represent the mean of three independent experiments. Asterisks indicate significant differences (***p <0.001).

**Figure S2. Lectin staining.** N2 and *pmr-1* nematodes were stained with FITC conjugated GNA and UEA lectins before infection. *n* = 10 for each sample. Scale bar = 100μm.
