# Supplementary figures and images for: Pmr-1 gene affects susceptibility of Caenorhabditis elegans to Staphylococcus aureus infection through glycosylation and stress response pathways' alterations
Source: Virulence. 2019 Nov 27;10(1):1013–25. doi: 10.1080/21505594.2019.1697118 (PMC6930020; doi:10.1080/21505594.2019.1697118)

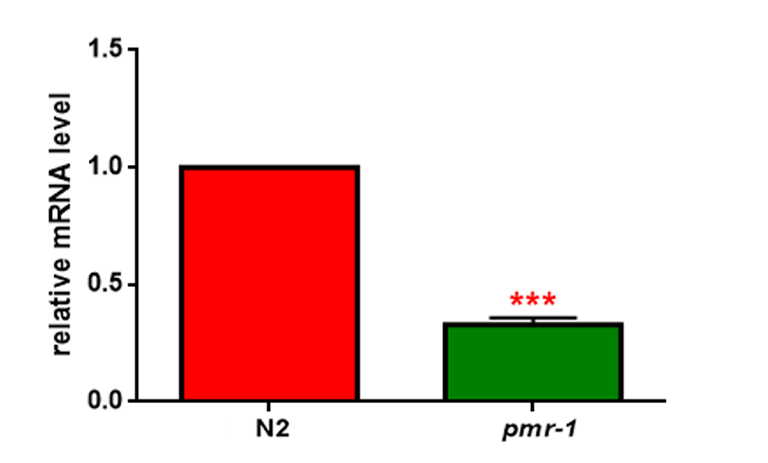

Supplement: Supplemental Material [file kvir-10-01-1697118-s001.zip › Figure_S1.tif]

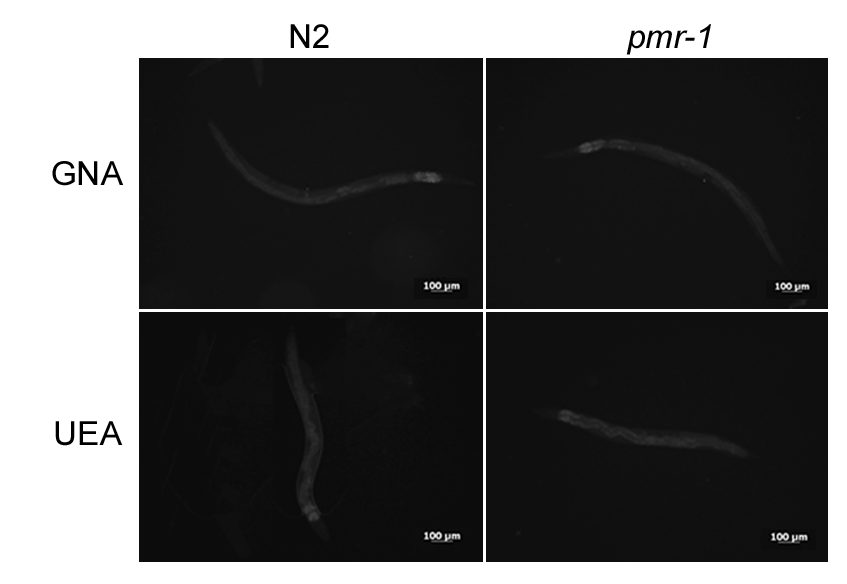

Supplement: Supplemental Material [file kvir-10-01-1697118-s001.zip › Figure_S2.tif]
